# Substantially enhanced rate capability of lithium storage in Na_2_Ti_6_O_13_ with self-doping and carbon-coating†

**DOI:** 10.1039/c8ra00468d

**Published:** 2018-02-28

**Authors:** Jin-Yun Liao, Taylor W. Smith, Raja R. Pandey, Xiaoqing He, Charles C. Chusuei, Yangchuan Xing

**Affiliations:** Department of Chemical Engineering, University of Missouri Columbia MO 65211 USA xingy@missouri.edu; Department of Chemistry, Middle Tennessee State University Murfreesboro TN 37132 USA; Electron Microscopy Core Facility, University of Missouri Columbia MO 65211 USA

## Abstract

Na_2_Ti_6_O_13_ (NTO) has recently been reported for lithium ion storage and showed very promising results. In this work, we report substantially enhanced rate capability in NTO nanowires by Ti(iii) self-doping and carbon-coating. Ti(iii) doping and carbon coating were found to work in synergy to increase the electrochemical performances of the material. For 300 cycles at 1C (1C = 200 mA g^−1^) the charge capacity of the electrode is 206 mA h g^−1^, much higher than that (89 mA h g^−1^) of the pristine NTO electrode. For 500 cycles at 5C the electrode can still deliver a charge capacity of 180.5 mA h g^−1^ with a high coulombic efficiency of 99%. At 20C the capacity of the electrode is 2.6 times that of the pristine NTO. These results clearly demonstrate that the Ti(iii) self-doping and uniform carbon coating significantly enhanced the kinetic processes in the NTO nanowire crystal, making it possible for fast charge and discharge in Li-ion batteries.

## Abstract 2

Ti^3+^ self-doping and carbon-coating are efficient approaches to simultaneously improve the rate capability and cyclability of Na_2_Ti_6_O_13_ nanowires for lithium storage.

## Graphical abstract

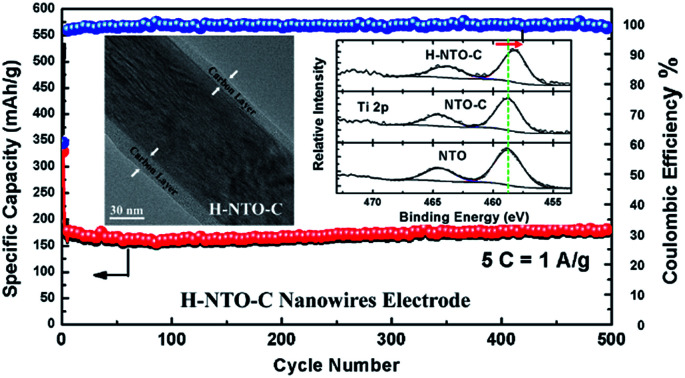

## 1. Introduction

As a Ti-based material, sodium titanate has been considered a promising anode material option in Li-ion batteries (LIBs) because of its low Li/Na-intercalation potential, wide availability, low cost, environmental friendliness, and ease of synthesis.^1–5^ Although recent research has shown that the smaller radius of Li^+^ means that it can be inserted into sodium titanate at a lower voltage of below 0.6 V (*vs.* Li^+^/Li),^6–8^ only a few reports have focused on sodium titanate as an anode material for LIBs. Most recently, Tian *et al.* reported the first use of layered Na_2_Ti_2_O_5_ hierarchical structures as anode materials for Li-ion batteries.^8^ The hierarchical structure of Na_2_Ti_2_O_5_ nanoparticles exhibited superior electrochemical performance. A capacity of 223.8 mA h can be maintained after 600 cycles at 50 mA g^−1^, and a capacity of 164.3 mA h g^−1^ can be achieved at a high current density of 1000 mA g^−1^. Na_2_Ti_6_O_13_ nanorods were reported by Shu *et al.* through a traditional solid state reaction and reported as anode materials for advanced lithium-ion batteries.^9^ The Na_2_Ti_6_O_13_ nanorods obtained at 900 °C only have a reversible capacity of 58 mA h g^−1^ at current density of 100 mA g^−1^. Na_2_Ti_6_O_13_ has been shown by Domiko *et al.* to accommodate up to three moles of lithium per mole of Na_2_Ti_6_O_13_. Lithium insertion into Na_2_Ti_6_O_13_ phase includes two solid solutions and a biphasic transition in the potential range between 1.0–1.5 V, and the process is very reversible. However, this material shows very poor cycling performance in Li-ion batteries. The capacity fading mostly is ascribed to the catalytic effect of directly exposed surface to the electrolyte.^6,10^

The main disadvantage of using sodium titanate as an LIB anode material is its low electronic conductivity, resulting in poor rate capability.^1,11^ Several approaches have been taken to improve the electronic conductivity of sodium/lithium titanate, such as electronic materials coating/mixing^3,12–14^ and ionic doping,^2,3,15,16^ but no carbon coating on sodium titanate as an anode material for Li-ion batteries has been reported. A uniform carbon coating layer on the surface of sodium titanate not only can improve the electrical conductivity, but also provide a protection barrier against possible electrolyte degradation on the surface of sodium titanate.

Herein, we introduce a carbothermal reduction technique which yields a uniform thin carbon coat; further hydrogenation treatment leads to Ti^3+^ self-doped monocrystalline Na_2_Ti_6_O_13_ nanowires with improved electronic conductivity suitable for LIB anode applications. Monocrystalline 1D structures and a highly conductive smooth carbon layer can significantly facilitate electron transportation along one-dimensional nanowires in the electrodes. When used as anodes for LIBs the hydrogenated, carbon-coated Na_2_Ti_6_O_13_ nanowires (H-NTO-C) manifest itself in much improved rate capability and superior cyclability.

## 2. Experimental section

### 2.1 Materials synthesis

Na_2_Ti_6_O_13_ was prepared by a hydrothermal synthesis. A 0.5 g amount of TiO_2_ (P25, Degussa) was first dispersed in 50 mL of 10 M NaOH aqueous solution, then transferred to a 100 mL Teflon-lined stainless steel autoclave and heated in an electric oven at 200 °C for 24 hours. After completion of the hydrothermal reaction, the white precipitate was filtrated and washed with an abundance of deionized water to remove the excess Na^+^. After being calcined at 500 °C for 5 hours in air, the resulting Na_2_Ti_6_O_13_ white powder was collected, and will subsequently be referred to as NTO, the pristine sample. Carbon-coated NTO (NTO-C) nanowires were prepared using a carbothermal coating process: 0.5 g prepared NTO powders were first placed in a tubular quartz furnace, and then thermally coated with carbon in 5% acetylene (C_2_H_2_) in N_2_ for 20 min at 600 °C.^17^ After carbon coating, the products were further annealed under 5% H_2_ in N_2_ at 600 °C for another 6 hours to achieve the final sample: Ti^3+^ self-doped and carbon-coated NTO nanowires (H-NTO-C).

### 2.2 Materials characterization

Powder X-ray diffraction (XRD) patterns were recorded using a PANalytical X'Pert Multi-Purpose Diffractometer with Cu Kα radiation in the 2*θ* range from 10° to 80°. A field emission scanning electron microscope (ESEM, FEI Quanta 600 FEG), transmission electron microscope (TEM, FEI F30), and high-resolution transmission electron microscope (HRTEM) were used to examine the morphologies and crystalline structures of the samples. The Raman spectra of NTO and NTO-C samples were collected on an InVia spectrometer equipped with a 20 mW diode laser, using an excitation wavelength of 632 nm and a spectrum range from 100 cm^−1^ to 2000 cm^−1^. The electron energy loss spectroscopy (EELS) in the TEM was used to get insights into the valence state of titanium ions before and after treatment in H_2_ atmosphere. The EELS spectrum were collected in the TEM image mode where collection semi-angle was determined by the objective aperture to be around 7 mrad and the sample areas for EELS measurements were defined by the entrance aperture in the Gatan imaging filter. X-ray photoelectron spectra (XPS) were acquired using a Perkin-Elmer PHI 560 system with a double-pass cylindrical mirror analyzer operated using a Mg Kα anode with a *hν* = 1253.6 eV photon energy operated at 250 watts and 13 kV. XPS peaks were curve-fitted using 70%-to-30% Gaussian–Lorentzian lineshapes with Shirley background subtractions.^18^ Binding energy peak envelopes were deconvoluted using CasaXPS ver. 2.2.107 (Devonshire, UK) software.

### 2.3 Electrochemical evaluation

The nanowire electrodes were fabricated with the active materials (NTO, NTO-C and H-NTO-C), carbon black (CB), and binder (PVDF, polyvinylidene fluoride) in the weight ratio 60 : 20 : 20 and using *N*-methyl-2-pyrrolidinone (NMP) as a solvent. This combination was mixed thoroughly, and the obtained slurry was coated uniformly on etched copper foil. The slurry-coated Cu foil was dried at 60 °C for one hour, followed by drying at 120 °C overnight in a vacuum oven. The dried electrodes were then cut into 11 mm diameter circular discs and pressed onto Al foil (at 10 kN) to ensure a close contact between the electrode materials and the current collector. The electrochemical characterization was performed using 2032-type coin cells with two-electrodes, assembled in an Ar-filled dry glove box using the prepared NTO-based electrodes and Li metal as the working electrode and counter electrode, respectively. A 1.0 M concentration of LiPF_6_ in ethylene carbonate (EC)/dimethyl carbonate (DMC) (1 : 1 by volume) was used as the electrolyte, and one piece of porous 25 μm thick polypropylene membrane (Celegard) was used as the separator. The discharge–charge cycling was performed between 0.01 and 2.5 V (*vs.* Li/Li^+^) at room temperature, using different C-rates from C/10 to 20C (1C = 200 mA g^−1^) on a battery tester (Arbin GT2000). Electrochemical impedance spectroscopy (EIS) was carried out in the frequency range of 1 MHz to 50 mHz on an electrochemical workstation (Camry Reference 3000), and the amplitude of the alternating voltage was 10 mV. The impedance parameters were determined by fitting of the impedance spectra using Z-view software. Cyclic voltammetry (CV) testing was performed between 0.01 and 3 V (*vs.* Li/Li^+^) with a scan rate of 0.1 mV s^−1^.

## 3. Results and discussion

### 3.1 XRD and TEM characterizations

**Fig. 1:**
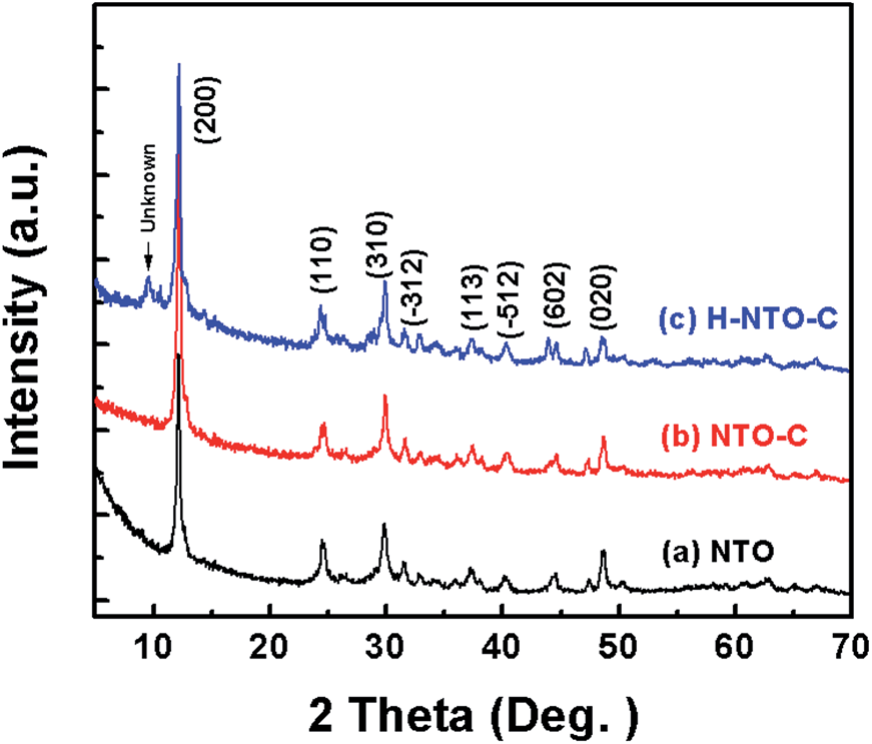
XRD patterns of the three prepared NTO based materials. (a) NTO, (b) NTO-C and (c) H-NTO-C.

Fig. 1 shows the XRD patterns of the three prepared sodium titanium oxide nanowire samples. Pattern (a) shows that the diffraction of the NTO sample clearly matches the sodium hexa-titanate (Na_2_Ti_6_O_13_) reference pattern (JCPDS73-1398) consistent with a space group *C*2/*m*.^8,19^ Although sodium hexa-titanate has been described as having a very similar structure to that of the tri-titanate, a different XRD pattern from the reported Na_2_Ti_3_O_7_ compound has been obtained by hydrothermal treatment using TiO_2_ and NaOH as reagents.^6,20^ The different phase of products using the same reagents may be due to differing experimental conditions or the crystalline phase of TiO_2_. Kolen'ko *et al.* reported the synthesis of nanorods of sodium tri-titanate and the transformation of this layered compound into three-dimensional frameworks of sodium hexa-titanate without significant changes to the particle morphology.^19^ Meng *et al.* studied the effect of the reagent phase using brookite and anatase TiO_2_,^21^ with the results showing that either of the two types of nanowires derived from the brookite or anatase phase can be indexed to the same phase of sodium hexa-titanate, but with obvious (200) diffraction peak shifted to a higher 2*θ* for anatase TiO_2_. The shift degree of the (200) diffraction peak to a higher 2*θ* position increased with increasing the NaOH/TiO_2_ molar ratio. Patterns (b) and (c) in Fig. 1 show the diffractions of NTO nanowires after carbon coating (NTO-C) and hydrogenation (H-NTO-C). It is readily observed that the diffraction peaks of the NTO-C and H-NTO-C nanowires have good agreement with those of the pristine NTO. The constant peak position indicates the phase structure of sodium hexa-titanate has not been altered during the carbon coating and further hydrogenation processes. However, an additional unknown diffraction peak occurring at around 2*θ* = 9 degrees was observed in the H-NTO-C sample, which warrants a further study in the future.

**Fig. 2:**
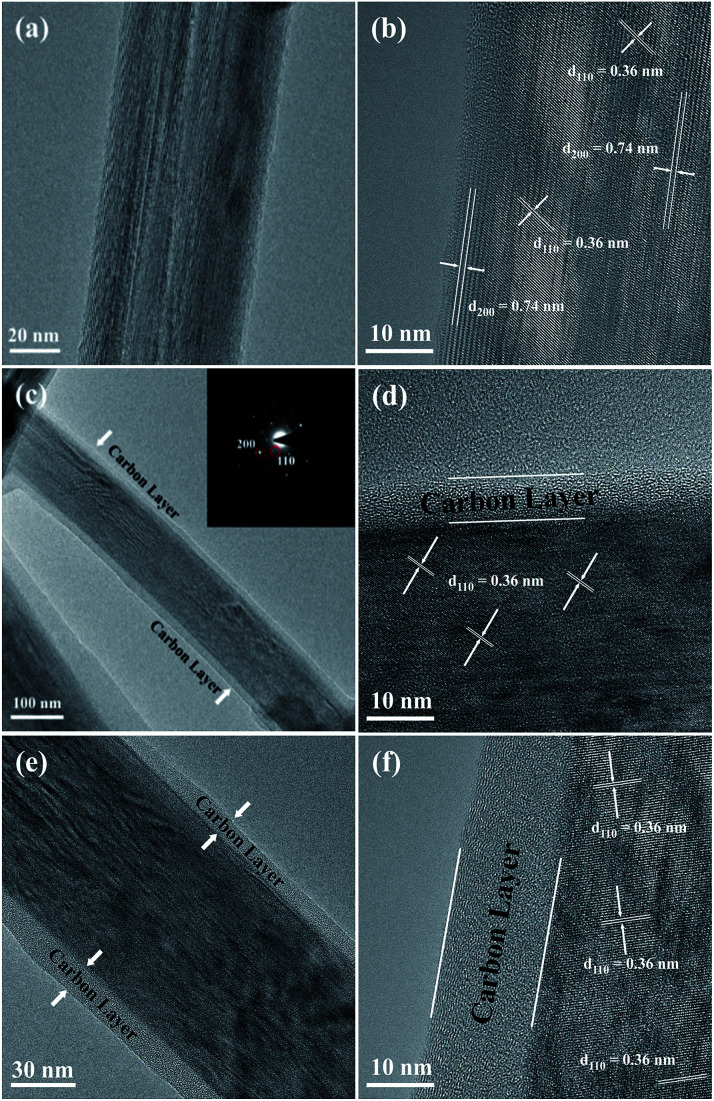
TEM and HRTEM images of the NTO-based nanowires. (a, b) NTO nanowire, (c, d) NTO-C nanowire (inset SAED pattern), (e, f) H-NTO-C nanowire.

The SEM images of the NTO, NTO-C, and H-NTO-C nanowires are shown in Fig. S1.† The diameters of the NTO nanowires determined from SEM imaging (Fig. S1(a)†) are about 50–300 nm, with lengths ranging from 5 to 20 μm. Consistent with the XRD results, the morphology of the nanowires remains unchanged after carbon coating treatment and further hydrogenation process according to the SEM images (Fig. S1(b) to (d)†). Further morphological and crystalline structure characterizations of the prepared NTO, NTO-C, and H-NTO-C nanowire materials were performed by TEM and high-resolution TEM (HRTEM), as shown in Fig. 2. Fig. 2(a) and (b) are standard TEM images of a single NTO nanowire, 65 nm in diameter, with the entire NTO nanowire clearly possessing a single crystalline structure oriented in the [100] direction. This can be further confirmed by the SAED pattern of NTO-C shown in Fig. 2(c) (inset). The interplanar spacing of *d*_200_ = 0.74 nm and *d*_110_ = 0.36 nm is in excellent agreement with the *d*-spacing of the sodium hexa-titanate (200) and (110) planes, respectively (JCPDS73-1398). After the carbon coating process, a larger nanowire with a diameter of around 350 nm was selected for TEM characterization as seen in Fig. 2(c) and (d). TEM revealed that the highly crystalline nature of the NTO nanowire is preserved according to the SAED pattern (inset of Fig. 2(c)) and clear lattice fringes shown in Fig. 2(d). The carbothermal reduction technique introduces a uniform layer of carbon coating with a ∼10 nm thickness onto the NTO nanowires. According to the TEM and HRTEM images of the H-NTO-C nanowire (Fig. 2(e) and (f)), no obvious changes were observed compared to the NTO-C nanowire. It is noted that the H-NTO-C sample still maintains the single crystal phase and uniform carbon coating for the nanowires.

The SEM images of the NTO, NTO-C, and H-NTO-C nanowires are shown in Fig. S1.† The diameters of the NTO nanowires determined from SEM imaging (Fig. S1(a)†) are about 50–300 nm, with lengths ranging from 5 to 20 μm. Consistent with the XRD results, the morphology of the nanowires remains unchanged after the carbon coating treatment and further hydrogenation process according to the SME images (Fig. S1(b) to (d)†). Further morphological and crystalline structure characterizations of the prepared NTO, NTO-C, and H-NTO-C nanowire materials were performed by TEM and high-resolution TEM (HRTEM), as shown in Fig. 2. Fig. 2(a) and (b) are standard TEM images of a single NTO nanowire, 65 nm in diameter, with the entire NTO nanowire clearly possessing a single crystalline structure oriented in the [100] direction. This can be further confirmed by the SAED pattern of NTO-C shown in Fig. 2(c) (inset). The interplanar space of *d*_200_ = 0.74 nm and *d*_110_ = 0.36 nm is in excellent agreement with the *d*-spacing of the sodium hexa-titanate (200) and (110) planes, respectively (JCPDS73-1398). After the carbon coating process, a larger nanowire with a diameter of around 350 nm was selected for TEM characterization as seen in Fig. 2(c) and (d). TEM revealed that the highly crystalline nature of the NTO nanowire is preserved according to the SAED pattern (inset of Fig. 2(c)) and clear lattice fringes shown in Fig. 2(d). The carbothermal reduction technique introduces a uniform layer of carbon coating with a ∼10 nm thickness onto the NTO nanowires. According to the TEM and HRTEM images of the H-NTO-C nanowire (Fig. 2(e) and (f)), no obvious changes were observed compared to the NTO-C nanowire. It is noted that the H-NTO-C sample still maintains the single crystal phase and uniform carbon coating for the nanowires.

### 3.2 Thermal stability analyses

Thermal analysis data of NTO-C nanowire materials is shown in Fig. S2,† including TGA trace and DTG curve. From the TGA trace, assuming that the final product is Na_2_Ti_6_O_13_, there is about a 5.73% total weight loss from room temperature to 700 °C. According to the derivative thermogravimetric (DTG) curve, there are three main peaks, at 40.7 °C, 102.3 °C and 387.6 °C, respectively. The first peak, observed from room temperature to 65.2 °C, is ascribed to desorption of surface free water (0.36% weight loss). The second peak, from 75.2 to 132.5 °C, represents desorption of chemically bonded water (0.85% weight loss). The largest weight loss occurs from 267 to 550 °C and is due to the coated carbon (4.52% weight loss). No noticeable weight loss is seen beyond 550 °C.

### 3.3 Spectroscopy analyses

**Fig. 3:**
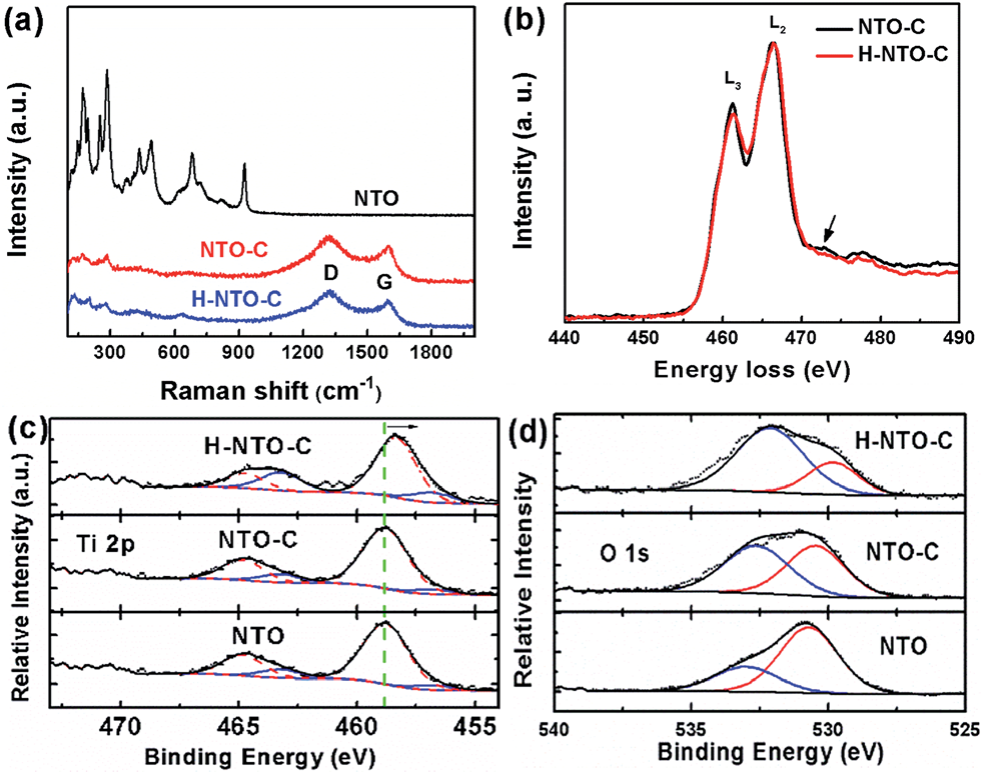
(a) Raman spectra of NTO, NTO-C and H-NTO-C nanowires, (b) electron energy loss spectroscopy (EELS) of NTO-C and H-NTO-C nanowires. XPS core levels of Ti 2p (c) and O 1s (d) orbitals of NTO, C-NTO and H-C-NTO samples.

Additional information on the composition and structural changes accompanying the carbon coating and hydrogenation processes for NTO-based nanowires was collected using Raman spectroscopy and EELS. The Raman spectra of all synthesized nanowires are depicted in Fig. 3(a). The lower frequency bands dominate the Raman spectrum of NTO nanowires. Generally, the Raman peaks below 400 cm^−1^ belong to Na–O bond vibrations, and bands between 600 and 800 cm^−1^ are attributed to the Ti–O stretching vibration in the edge-shared TiO_6_ octahedra.^22–24^ The bands over 800 cm^−1^ are ascribed to the short, low coordination Ti–O bonds involving non-bridging oxygen atoms. In the typical structure of Na_2_Ti_3_O_7_, every oxygen atom is coordinated to one titanium atom with a short Ti–O bond distance of 1.7 Å, resulting in a high frequency Raman peak at around 890 cm^−1^.^22^ For the sodium hexa-titanate (Na_2_Ti_6_O_13_) prepared in this work, each oxygen is bound to at least two titanium atoms. This high coordination will reduce the bond distance as low as 1.14 Å.^25^ Most likely, the short Ti–O bond distance is what gives rise to the highest frequency at 928 cm^−1^. After carbothermal reduction, the color of the NTO powder changed from white to black, and two additional peaks were observed in the NTO-C and H-NTO-C Raman spectra, attributed to the D-band (*ca.* 1325 cm^−1^) and G-band (*ca.* 1604 cm^−1^) of the carbon material, respectively.^14^ Two typical EELS spectra were collected from the two set of samples (NTO-C and H-NTO-C) under the same imaging conditions and were normalized with respect to L_2_ peaks after background subtraction (Fig. 3(b)). It is seen that the L_3_ peak becomes weaker after treatment, suggesting that the valence of Ti is decreased. It was reported that the L_2_/L_3_ intensity ratio can be used to determine the valence state of 3d transitional metals and it gets higher when valence becomes larger.^26–28^ In addition, the small bump highlighted by the arrow in the electron energy-loss near edge fine structure (ELNES) region indicated that the surrounding environment of the Ti atoms changes as well after the treatment, most likely due to the formation of oxygen vacancies.

The valence change in Ti has been further confirmed by XPS analysis shown in Fig. 3(c) and (d) and S3.† Fig. S3† summarizes the XPS data with stack plots for the C 1s, O 1s, Na 1s, and Ti 2p core levels of the Na_2_Ti_6_O_13_ compound from which these three precursors had been synthesized: untreated compound (NTO), the carbon-coated compound (NTO-C), and the treated compound after hydrogenation (H-NTO-C).

As shown in Fig. 3(c), Ti 2p core levels for the NTO and NTO-C remained relatively constant at *ca.* 457 and *ca.* 464 eV for the 2p_3/2_ and 2p_1/2_ orbitals, respectively, matching the literature for TiO_2_.^29^ The slight decrease in overall binding energy (BE) of the envelopes for these orbitals observed when the sample was hydrogenated (H-NTO-C), consistent with an increase in electron density within the Ti atoms, in which a very small amount of the Ti^4+^ is reduced to Ti^3+^ upon hydrogen treatment at 600 °C.

Further deconvolution using, CasaXPS ver. 2.2.107 software (Devonshire, UK), of the Ti 2p orbitals was achieved by constricting peak centers (with full-width-at-half-maxima in parentheses) at 456.8 (2.0) eV and 463.2 (2.0) eV to the Ti^3+^ chemical oxidation state [a] as a guide, respectively (Fig. 3(c)), and allowing the remaining peaks to be optimized within the CasaXPS curvefitting algorithm. Additional Ti 2p BE peaks, denoting Ti^4+^ were as follows. For NTO, additional Ti 2p peaks were found at 458.8 (2.4) and 464.8 (2.4) eV; for NTO-C they were at 458.8 (2.2) and 464.8 (2.0) eV; and for H-NTO-C, they were at 458.3 (2.0) and 464.7 (1.9) eV. Ratios of the Ti^3+^-to-Ti^4+^ were quantified using the integrated peak areas of the deconvoluted Ti 2p spectra. With these curvefitted parameters, the Ti^3+^ : Ti^4+^ of the NTO, NTO-C and H-NTO-C were 0.191, 0.145 and 0.380, respectively. This result confirms the presence of species of Ti^3+^ ions or oxygen vacancies in the H-NTO-C nanowires, which increased with hydrogenation, consistent with the EELS results above.

The O 1s levels (Fig. 3(d)) at 530.5–530.7 eV for NTO and NTO-C is elevated from the typically expected BE for TiO_2_ oxygen (at 530.0 eV).^30^ We assign this higher O 1s BE state to Na_2_Ti_6_O_13_ oxygen. Additional, accompanying oxidation states at 533.0 eV for NTO and 532.6 eV for NTO-C, denote adsorbed H_2_O^31^ and adsorbed O-containing moieties, possibly due to polymeric groups^32^ on these surfaces, respectively. For H-NTO-C, the lower O 1s oxidation state at 529.8 eV matches the expected literature value for TiO_2_;^30,33^ the higher O 1s BE at 532.1 eV is attributed to an adsorbed O-containing surface species. A trend is observed in which the metal oxide state at *ca.* 530 eV decreases with respect to adsorbed O-containing species as treatment ensues: NTO → NTO-C → H-NTO-C.

### 3.4 Electrochemical performances

**Fig. 4:**
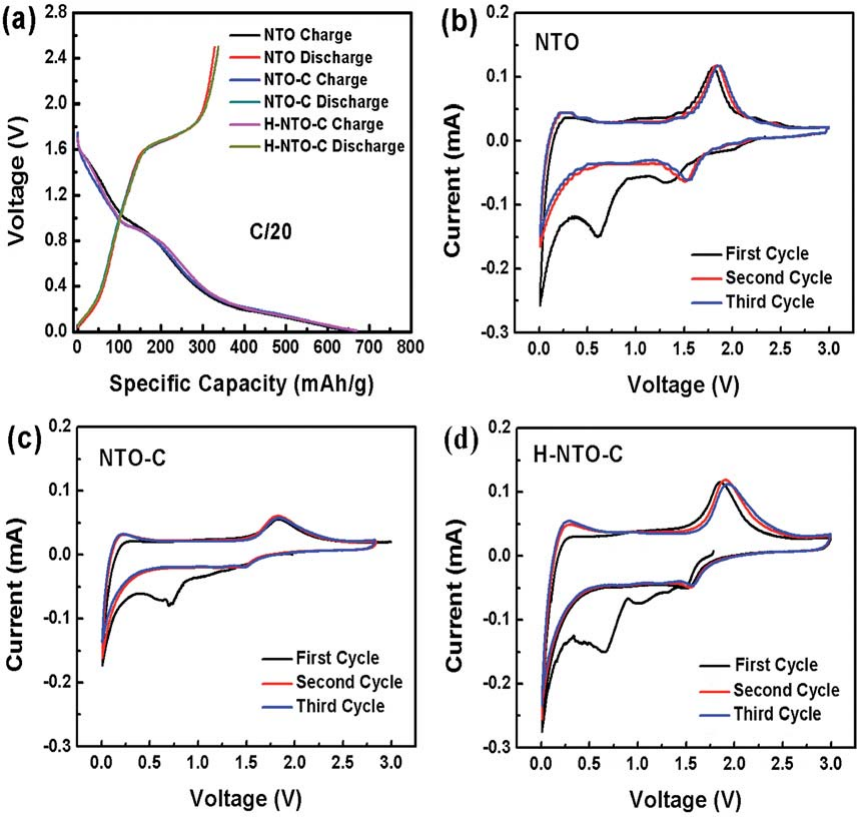
(a) Comparison of (a) discharge–charge curves and CV curves of (b) NTO, (c) NTO-C and (d) H-NTO-C nanowire electrodes.

In order to evaluate the electrochemical performance of the prepared NTO-based nanowire materials, NTO, NTO-C, and H-NTO-C nanowire anodes were assembled in half-cells utilizing metallic lithium film as the counter electrode. The first discharge–charge cycle profiles for the three NTO-based nanowire electrodes are shown in Fig. 4(a) (0.01 to 2.5 V, at a rate of C/20). All three electrodes delivered similar initial discharge and charge capacities of around 330 mA h g^−1^ and 660 mA h g^−1^, respectively. These large irreversible capacities observed over the first cycle may be attributed to: (i) the formation of a solid electrolyte interphase (SEI) layer on the NTO-based nanowires due to electrolyte decomposition; (ii) irreversible intercalation of Li^+^ ions into Na_2_Ti_6_O_13_; or (iii) the presence of trace water on the electrode material surface or within the electrode, with physically adsorbed water and crystalized water generating oxygen during cycling to consume irreversibly a certain amount of lithium over the initial cycle.

The electrochemical insertion of lithium into Na_2_Ti_6_O_13_ above 0.6 V can be described by Na_2_(Ti^4+^)_6_O_13_ + *x*e^−^ + *x*Li^+^ → Na_2_Li_*x*_(Ti^3+^)_6_O_13_, and the slope from 0.01 to 0.4 V is ascribed to further lithium intercalation into the layered Na_2_Li_*x*_Ti_6_O_13_.^6–10^Fig. 4(b)–(d) shows the CVs for NTO, TNO-C and H-NTO-C anodes between 0.01 and 3 V (*vs.* Li/Li^+^). During the initial negative scan, there is a broad peak observed at around 0.7 V, which then disappears in the following cycles. This result is in good agreement with the low coulombic efficiencies at first cycle calculated in the discharge/charge profiles (Fig. 4(a)). In subsequent cycles, a pair of cathodic/anodic peaks located at around 1.56 V and 1.8 V appear, characteristic of lithium ion intercalation/deintercalation in the monoclinic Na_2_Ti_6_O_13_. The potential differences between the redox peaks of NTO, NTO-C, and H-NTO-C electrodes are 370 mV, 350 mV, and 340 mV, respectively, indicating gradually reduced polarization of the electrode associated with Li^+^ intercalation/deintercalation after carbon coating and hydrogen treatment.

**Fig. 5:**
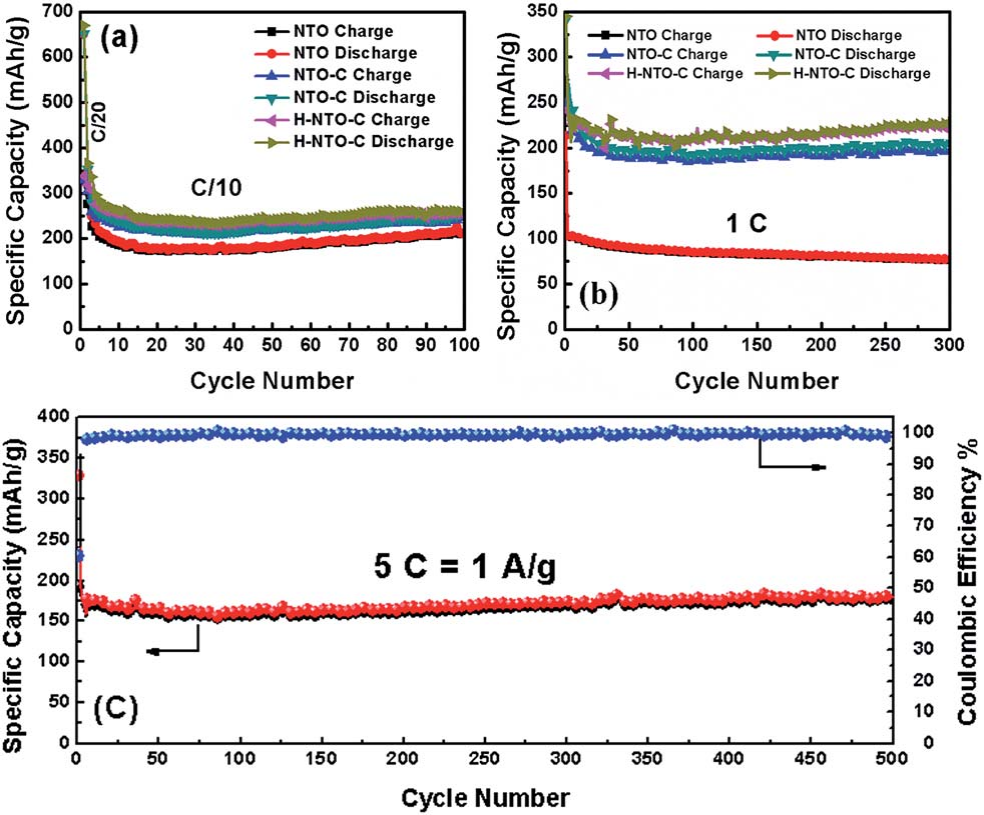
Comparison of cycling performance of NTO, NTO-C, and H-NTO-C nanowire electrodes at (a) C/10 and (b) 1C. (c) The cyclability of H-NTO-C at high C-rate of 5C up to 500 cycles.

The cyclability and rate capability of the NTO-based electrodes in Li-ion batteries are further examined as shown in Fig. 5 and 7. Fig. 5 compares the cycling performances of the three NTO-based nanowire electrodes at C/10, 1C, and 5C, respectively, in a voltage range between 0.01 V and 2.5 V. As seen in Fig. 5(a), the H-NTO-C electrodes showed the highest specific capacity, followed by NTO-C, and then NTO. After the first two formation cycles at C/20, the charge capacity of NTO, NTO-C and H-NTO-C electrodes are 210, 248, and 255 mA h g^−1^ after 100 cycles cycling at C/10, showing 92%, 94% and 95% retention of charge capacity, respectively. The higher capacity retention for the NTO-C and H-NTO-C electrodes is due to the presence of the uniform carbon layer on the surface of the NTO nanowires, providing mechanical protection and stabilizing the SEI layer at lower potentials.^7,9,34^ Moreover, when these electrodes were measured at 1C over 300 cycles (Fig. 5(b)), the H-NTO-C electrode showed a much higher specific capacity (231 mA h g^−1^, 95% of initial charge capacity) than the pristine NTO electrode (75 mA h g^−1^, 73% of initial charge capacity). Both treatments can improve the electrical conductivity, providing fast electron/ion transportation along the one-dimension nanowires.^1,3,4^ More substantially, the H-NTO-C nanowire electrode maintained a capacity of 178 mA h g^−1^ (92% of initial charge capacity) with a coulombic efficiency of over 99% (Fig. 5(c)) after cycling at a 5C rate for 500 cycles.

**Fig. 6:**
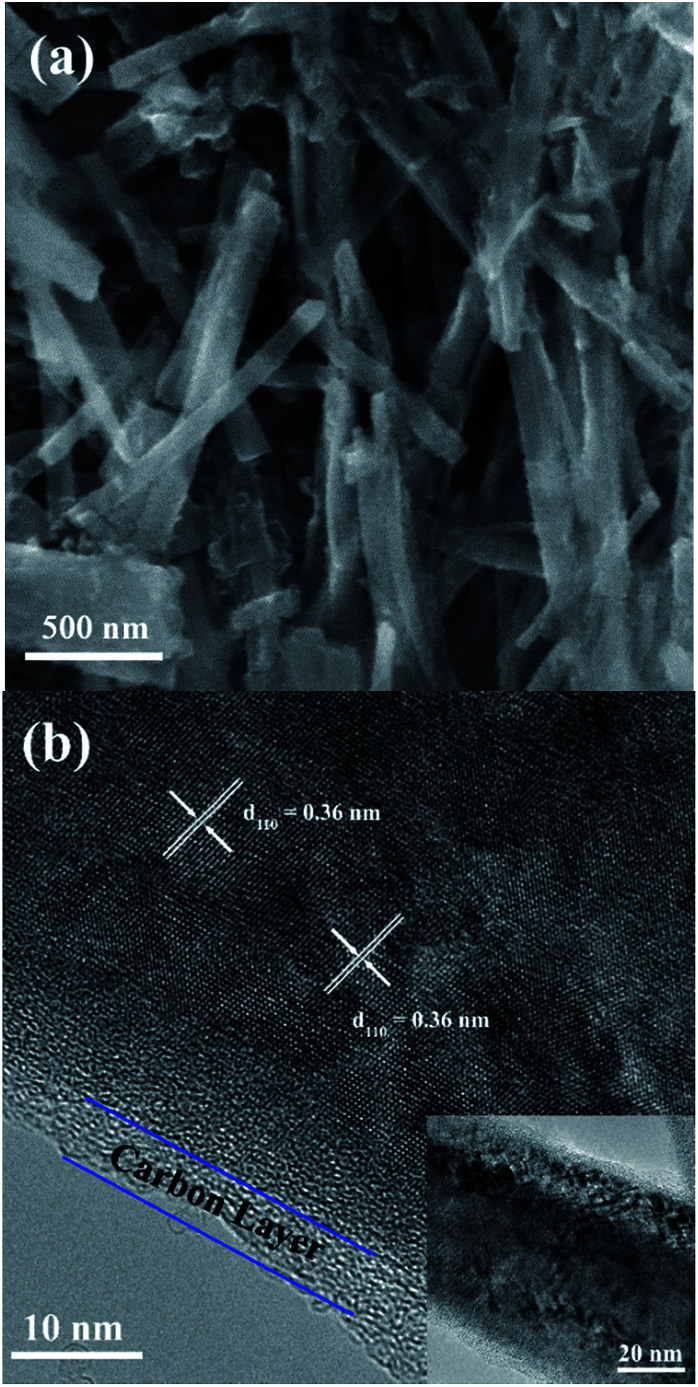
*Ex situ* SEM (a) and TEM (b) images of cycled H-NTO-C nanowires after 300 cycles at 1C.

The morphological evolutions of the NTO-based nanowire electrodes after 300 discharge/charge cycles at 1C are shown in Fig. 6 and S4.† Compared with the pristine NTO, it appears that only negligible volume expansion has occurred for the three nanowire materials,^1^ and the nanowire morphology is also maintained (Fig. 6(a)). The interplanar space of *d*_110_ = 0.36 nm remains in agreement with the *d*-spacing of the sodium hexa-titanate (110) planes. As seen in Fig. 6(b), the high resolution TEM of the cycled H-NTO-C nanowire shows the crystal structure and coated thin carbon layer remain intact even after extensive cycling. The negligible volume expansion and crystalline structure change during intercalation are critical for cycling stability of electrodes.

**Fig. 7:**
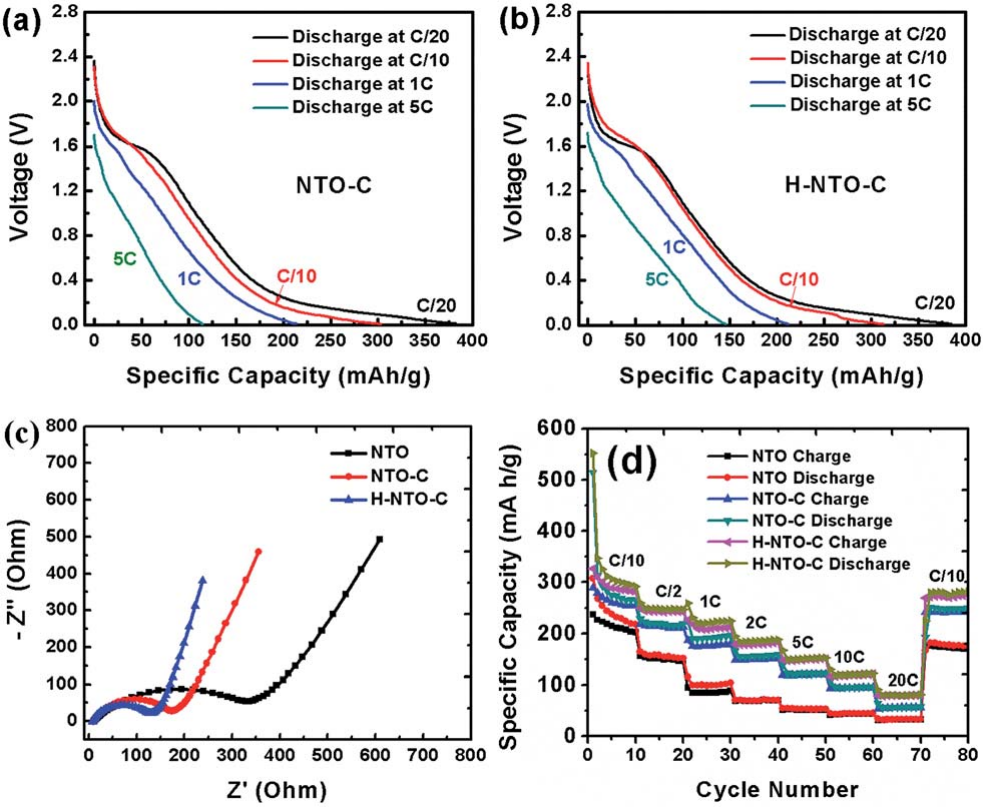
Rate capabilities of the NTO-based nanowire electrodes. Galvanostatic profiles of (a) NTO-C and (b) H-NTO-C nanowire electrodes at various current rates. (c) Nyquist plots and (d) rate cycling performances of NTO, NTO-C and H-NTO-C nanowire electrodes.

Rate capability is another very important consideration for practical LIBs, especially for use in electric vehicles. The discharge curves of NTO-based electrodes at different rates are shown in Fig. 7 and S5.† As the current density increases, all discharge voltage slopes shift gradually toward low voltage, indicating an increased electrode polarization at high current densities. However, when compared to the NTO electrode (Fig. S5a†), higher capacity and smaller electrode polarization were observed for both NTO-C (Fig. 7(a)) and H-NTO-C (Fig. 7(b)) electrodes, indicative of that uniform carbon coating and Ti^3+^ self-doping processes can significantly improve the conductivity of as-prepared NTO nanowires. In addition, EIS measurements were carried out to investigate the conductivity of the nanowire electrodes. Nyquist plots of the three electrodes before cycling are shown in Fig. 7(c), S6 and Table S1,† with the charge-transfer resistances of the NTO, NTO-C, and H-NTO-C nanowire electrodes measuring 343, 170, and 120 ohms, respectively.

The large decrease in charge-transfer resistance from NTO to NTO-C electrodes (from 343 to 170 ohms) further supports the earlier conclusion that the uniform carbon layer on the NTO nanowire significantly enhances the nanowire conductivity. Additional hydrogen treatment on NTO-C nanowires can further reduce the ohmic resistance of the H-NTO-C electrode, meaning that even lower activation energy is required for Li^+^ diffusion. The faster transfer rate of Li ions in the H-NTO-C nanowire electrode will deliver a superior rate capability *versus* NTO and NTO-C electrodes. To examine the effect of carbon coating and Ti^3+^ self-doping on rate capability, all three nanowire electrodes were discharged/charged at different rates from C/10 to 20C and then back to C/10, as shown in Fig. 7(d). Upon comparison of the NTO electrode at different discharge/charge rates with the other two treated materials, the H-NTO-C with both carbon coating and Ti^3+^ self-doping demonstrates the best performance, especially at high C-rate. Compared to the pristine NTO electrode, the H-NTO-C electrode shows around 21% capacity improvement at a rate of C/10. However, when cycling at 20C, the H-NTO-C electrode exhibits capacity improvement as high as 161%. Specifically, the H-NTO-C electrode demonstrated the best rate performance of the three samples tested, as shown in Fig. S6(b),† with charge capacities of 278, 147 and 84 mA h g^−1^ at C/10, 5C and 20C, respectively. Meanwhile, the pristine NTO nanowire electrode only delivered charge capacities of 228, 56 and 32 mA h g^−1^ at C/10, 5C and 20C, respectively. The greatly improved rate capability of the H-NTO-C nanowire electrode can be primarily attributed to its high electronic conductivity arising from the uniform thin carbon layer and Ti^3+^ self-doping on/in NTO crystal structure, providing fast electron transport along the one-dimensional structure throughout the electrode. After high-rate measurement, the H-NTO-C nanowire electrode was still able to recover 96% of its charge capacity once the current rate was set back to C/10, better than either the NTO (85% capacity retention) or NTO-C (94% capacity retention) electrodes.

## 4. Conclusions

In summary, we have for the first time designed and fabricated Ti^3+^ self-doped and carbon-coated Na_2_Ti_6_O_13_ nanowires *via* a hydrothermal approach, combined with carbothermal coating and hydrogenation. Their electrochemical properties were investigated for use as anode materials in LIBs. The uniform carbon layer on the nanowire surface and Ti^3+^-doped Na_2_Ti_6_O_13_ crystal structure simultaneously enhance the electronic conductivity and surface electrochemical activity of the host material. The H-NTO-C nanowire electrode exhibits much enhanced electrochemical performances compared to the as prepared NTO nanowire electrode, especially in rate capability. When cycling at C/10, the H-NTO-C electrode shows a 21% capacity improvement compared to the pristine NTO electrode from 228 to 278 mA h g^−1^. However, when the cycling rate is increased to 20C, the H-NTO-C electrode capacity more than doubles, jumping from 32 to 84 mA h g^−1^ (a 161% increase). For long-term cycling at 1C, the H-NTO-C nanowire electrode can maintain a charge capacity of 206 mA h g^−1^ after 300 cycles, much higher than the values of 89 mA h g^−1^ observed for the same cycled NTO electrode. In addition, after operating the H-NTO-C nanowire electrode at 5C for 500 cycles it still can deliver a charge capacity of 180.5 mA h g^−1^ (94.1% of initial charge capacity), with a high coulombic efficiency of around 99%. This work illustrates that carbothermal reduction and hydrogenation processes are an effective technique for preparing highly conductive nanowires, and that these materials show great promise for use in high rate anodes for high power density LIB applications.

## Conflicts of interest

The authors declare no conflicts of interest.

## Supplementary Material

RA-008-C8RA00468D-s001
