# Supplementary material for: Substantially enhanced rate capability of lithium storage in Na2Ti6O13 with self-doping and carbon-coating
Source: RSC Adv. 2018 Feb 28;8(16):8929–36. doi: 10.1039/c8ra00468d (PMC9078603; doi:10.1039/c8ra00468d)
Supplement: RA-008-C8RA00468D-s001 [file RA-008-C8RA00468D-s001.pdf]

Electronic Supplementary Material (ESI) for RSC Advances.
This journal is © The Royal Society of Chemistry 2018

Electronic Supplementary Information

Substantially Enhanced Rate Capability of Lithium Storage in $Na_2Ti_6O_{13}$ with Self-doping and Carbon-coating

Jin-Yun Liao,[a] Taylor W. Smith,[a] Raja R. Pandey,[b] Xiaoqing He,[c] Charles C. Chusuei[b] and Yangchuan Xing[a]*

[a]Department of Chemical Engineering, University of Missouri, Columbia, Missouri 65211

[b] Chemistry Department, Middle Tennessee State University, Murfreesboro, TN 37132

[c] Electron Microscopy Core Facility, University of Missouri, Columbia, Missouri 65211

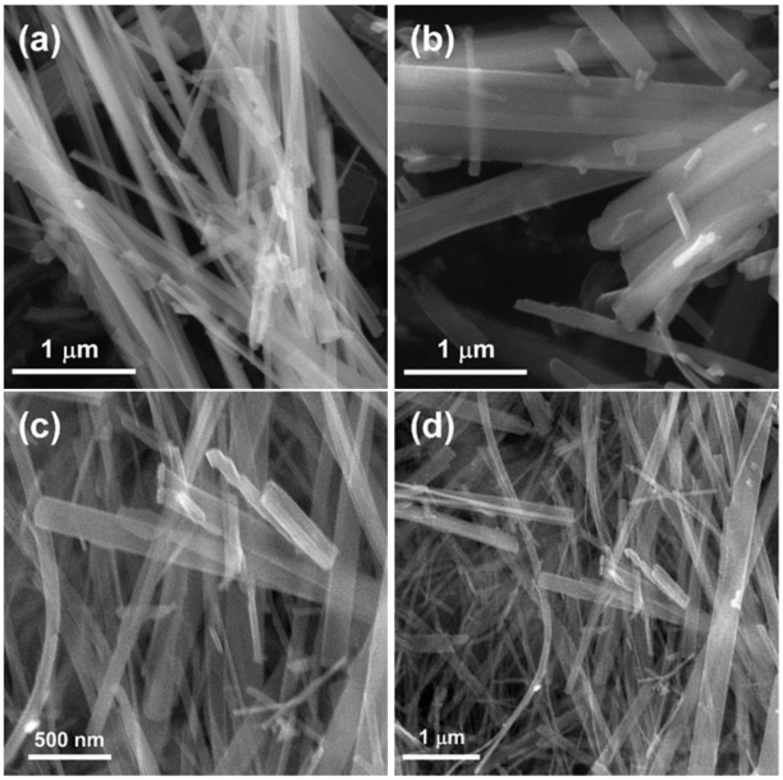

Fig. S1 SEM images of prepared NTO-based nanowires. (a) Pristine NTO nanowires, (b) carbon coated NTO-C nanowires and (c, d) Ti^{3+} self-doped and carbon coated H-NTO-C nanowires.

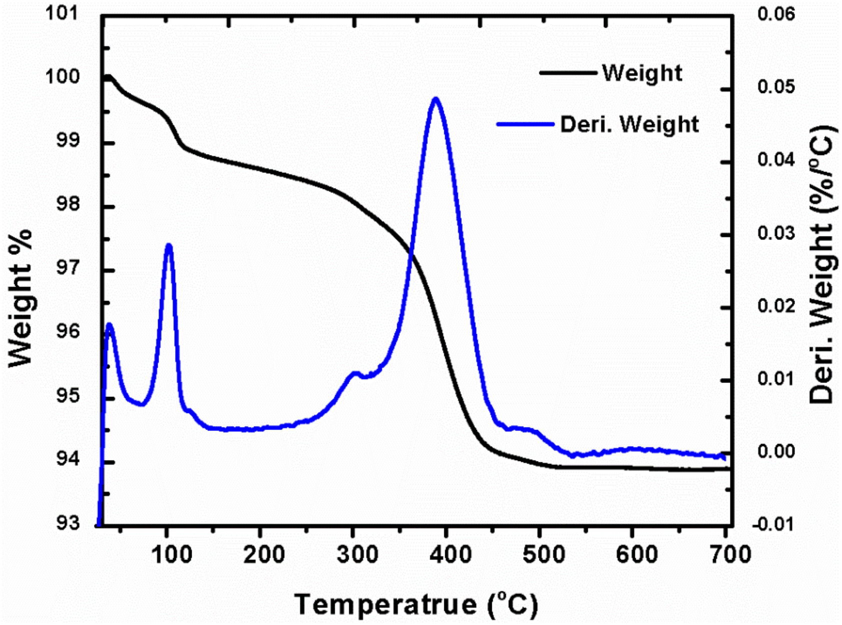

Fig. S2 TGA curve of carbon coated NTO-C nanowires.

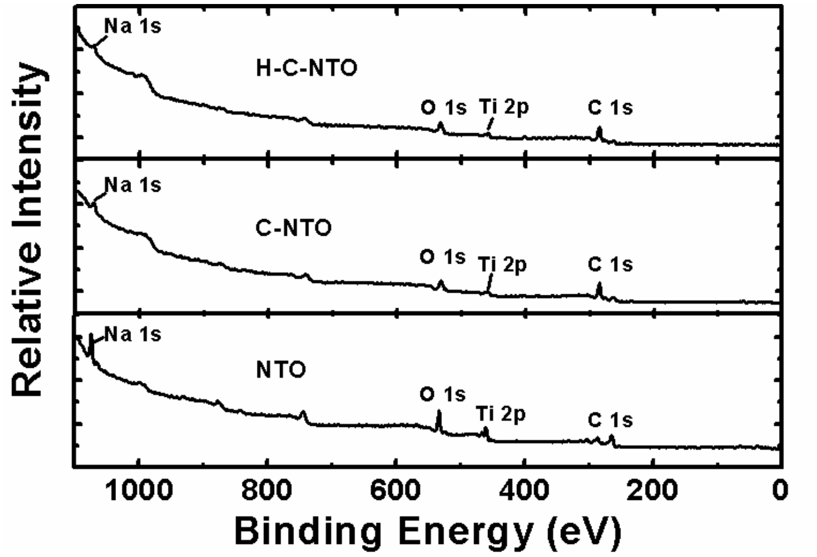

Fig. S3 Survey scan of NTO, C-NTO and H-C-NTO samples.

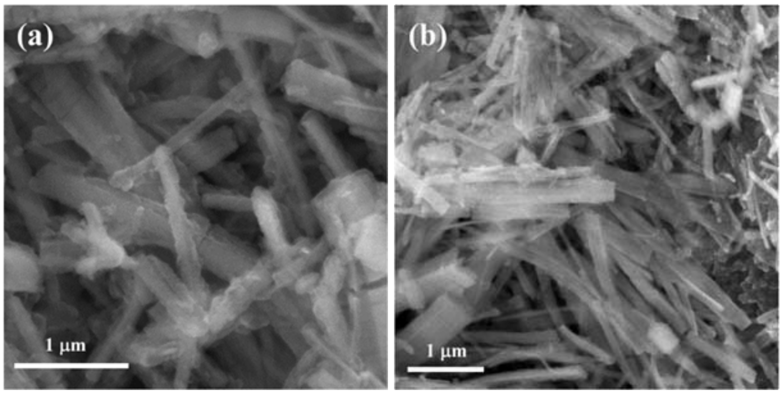

Fig. S4 SEM images of (a) NTO and (b) NTO-C electrodes after 300 cycles at 1C rate in Li-ion batteries.

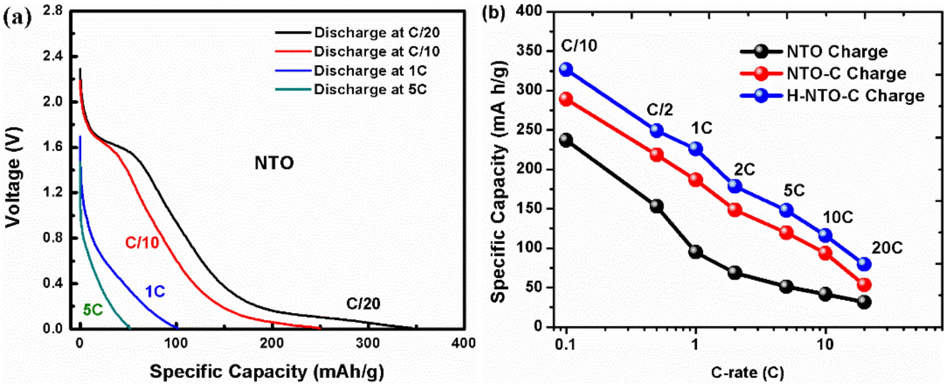

Fig. S5 Galvanostatic profiles of (a) NTO electrode at various current rates. (b) Rate capability comparison of NTO, NTO-C and H-NTO-C electrodes.

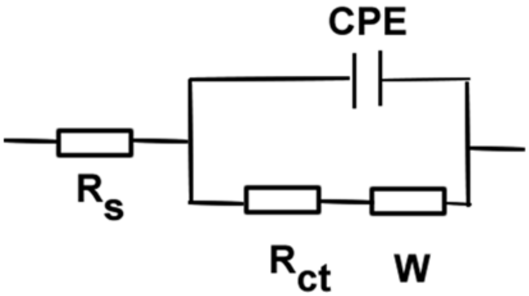

Fig. S6 Selected equivalent circuit used to fit the EIS Nyquist plots. The Rs reflects electric conductivity of the electrolyte, separator, and electrodes, which corresponded to the first semicircle at high frequency; Rct and Cdl were the charge transfer resistance and its relative double-layer capacitance, which corresponded to the second semicircle at medium frequency; W was the Warburg impedance related to the diffusion effect of Li, which was generally indicated by a sloping line at low frequency. The fitted Rs and Rct are listed in Table S1.

Table S1. Rs and Rct fitted by Z-view with selected equivalent circuit (Fig. S6)

Sample	Rs (Ohm)	Rct (Ohm)
NTO	7.2	343
NTO-C	6.9	170
H-NTO-C	6.7	120
